# Strategies for Coping With Stress in Athletes During the COVID-19 Pandemic and Their Predictors

**DOI:** 10.3389/fpsyg.2021.624949

**Published:** 2021-03-02

**Authors:** Marta Szczypińska, Aleksandra Samełko, Monika Guszkowska

**Affiliations:** ^1^Faculty of Physical Education, Józef Piłsudski University of Physical Education in Warsaw, Warsaw, Poland; ^2^Faculty of Rehabilitation, Józef Piłsudski University of Physical Education in Warsaw, Warsaw, Poland

**Keywords:** pandemic, COVID-19, athletes, coping, stress, Tokyo 2020

## Abstract

The aim of the study was to compare the strategies of coping with stress during the COVID-19 epidemic in athletes involved in Olympic preparations (57 potential Olympians) and students of physical education (54 extramural students), and to determine their depending on the variable gender. The research was conducted in the form of an on-line survey in the period of April 7–28 during the COVID-19 pandemic. Four standard psychological questionnaires were used. Elite athletes and physical education students practicing sports most often dealt with the stress of the COVID-19 pandemic using cognitive and behavioral coping strategies. The sports level depended on the strategies of coping with the stress of the COVID-19 pandemic more strongly than gender. The relationship between the sense of coherence (mainly comprehensibility) and the hope for success treated as a generalized immune resource with coping strategies in the case of the COVID-19 pandemic postulated by Antonovsky was confirmed.

## Introduction

The SARS-CoV-2 coronavirus infectious disease COVID-19 pandemic began on November 17, 2019 in Wuhan city, central China, and on March 11, 2020 was designated a pandemic by the World Health Organization (WHO) (World Health Organization, 2020). By July 8, 2020, over 11.80 million cases of COVID-19 had been reported in 188 countries and territories, including nearly 544,000 deaths and over 6.35 million recoveries (Johns Hopkins University, 2020). From the point of view of the psychology of stress, the pandemic phenomenon can be treated as a stressor. It belongs to the category of stressors affecting large groups of people, such as natural disasters (Lazarus and Cohen, 1977; Lepore and Evans, 1996) and chronic, but not acting continuously (Elliot and Eisdorfer, 1982).

A pandemic is a universal stressor, as it threatens the health and life of all people (Norris et al., 2002). It causes a feeling of helplessness and the loss of a fundamental sense of security, prevents the satisfaction of many basic needs, including protection, stability and the ability to predict one's own future (Bonanno et al., 2007; Shigemura et al., 2020). Athletes are just as vulnerable as the general population to the negative psychological consequences of COVID-19, such as stress, anxiety and depression (Mehrsafar et al., 2020).

On an international scale, measures have been taken to prevent the spread of disease. In 177 countries, schools and universities have been closed at the national or local level, which globally affected nearly 1.27 billion pupils and students (72.4%) (UNESCO, 2020). These actions have made the pandemic a source of additional stress for students, including at the university level. University students had to adapt to the changes resulting from new forms of remote learning, limited access to information sources and remote obtainment of knowledge. Some of them were deprived of opportunities of earning money, while many were forced to return to their family home (UNESCO, 2020).

The athletes who had to stop their daily organized training almost overnight also experienced additional stress. Closure at home, limitation of previous physical activity, isolation from members of sports teams and the sports community, and lack of social support negatively affected their psychophysical condition (Mehrsafar et al., 2020). In many countries, including Poland, the use of sports facilities has been banned and the activities of sports teams and clubs have been suspended. Athletes, including students of physical education involved in sports activities, were thus confronted with additional stressors resulting from the suspension of organized training (Pillay et al., 2020).

The COVID-19 pandemic caused the greatest disruption to the world sports calendar since World War II. Sporting events have been canceled or postponed worldwide (BBC, 2020; Los Angeles Times, 2020a). On March 24, 2020, the Organizing Committee of the IOC and Tokyo announced that the 2020 Summer Olympic and Paralympic Games would be postponed and would be held no later than summer 2021. For the first time in the history of the modern Olympic Games, their dates have been postponed (BBC Sport, 2020; Los Angeles Times, 2020b). The elite athletes who were preparing for the Olympic Games found themselves in an exceptionally difficult psychological situation. Participation in the Olympic Games is usually the most important event in a sporting career, and an Olympic nomination is a particularly important long-term goal for many athletes. The postponement of the Olympics put into question the participation in this event. This can be expected to increase the stress of the pandemic.

According to Antonovsky (1987), whose salutogenetic concept is the theoretical basis of our research, stressors are the requirements of the environment, for which there are no ready or automated adaptive reactions. They generate states of emotional tension, the content of which, according to the relational approach, will depend on the cognitive assessment of the situation (Lazarus and Folkman, 1984). In the case of assessing the situation as unfavorable (burdensome, exceeding resources, and threatening well-being), the subject experiences a state of stress, which consists of strong, most often negative emotions (fear, anxiety, and anger), less often hope and the accompanying physiological and biochemical changes exceeding the basic level of arousal (Strelau, 2000).

**Figure 1:**
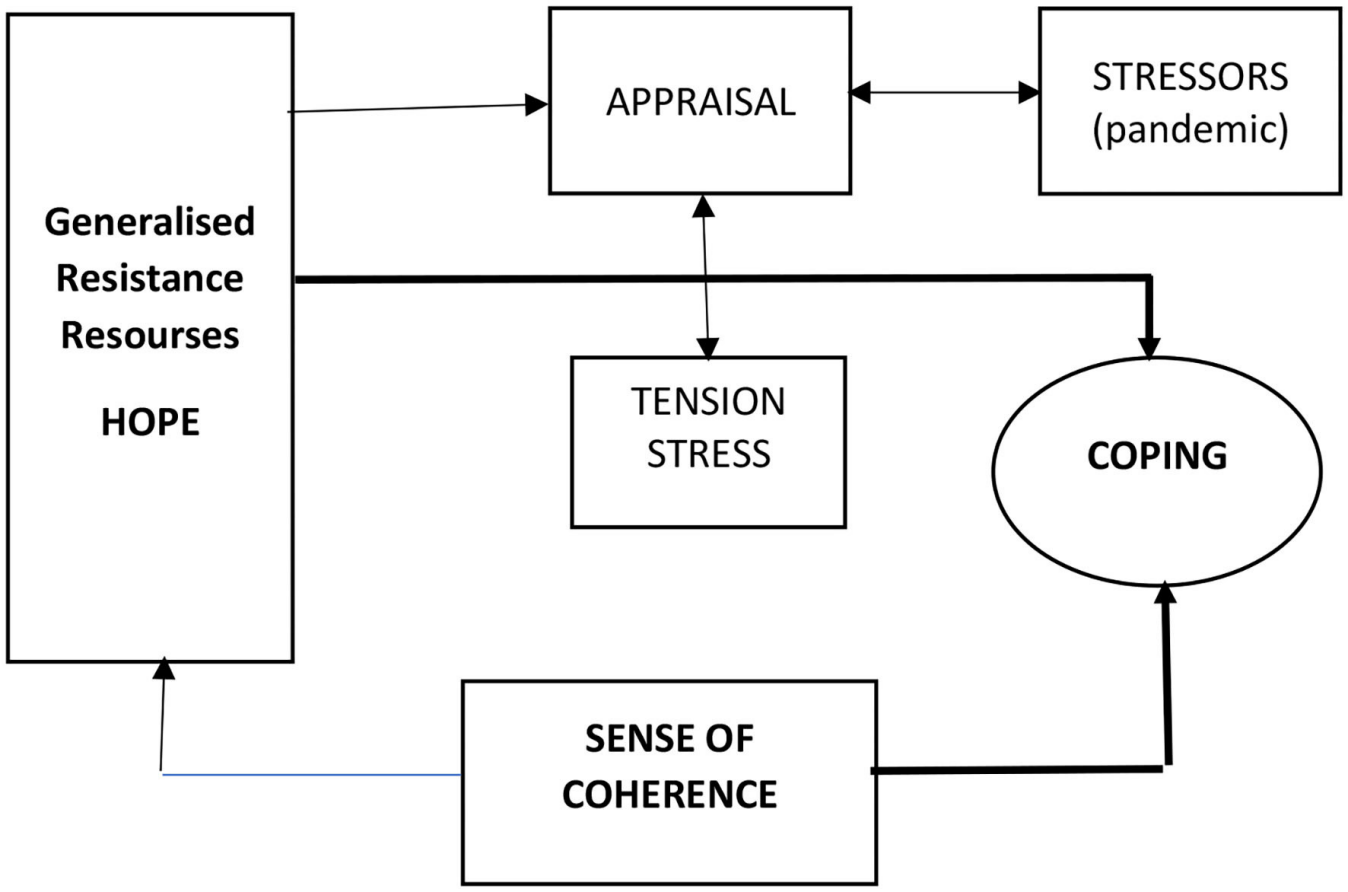
Studied variables in the model of salutogenesis (Antonovsky, 1979).

According to Antonovsky (1979) (Figure 1), it is not only stressors that determine the health costs of a stress relationship. Generalized immune resources and a sense of coherence are also important. Generalized immune resources are properties of an individual or collective entity that enable them to avoid stressors or (when it is impossible) to cope with the tension they generate (Pasikowski, 2000). They affect how stressors are assessed and how much stress they cause, and how the individual copes with stress (Antonovsky, 1987). One such resource may be hope, understood as the belief that one has the competence to achieve success. This construct, proposed by Snyder (Snyder et al., 1997, 2000), consists of two related beliefs. The first is the belief that the individual is able to implement the adopted plan (the belief that they can initiate the pursuit of the goal and persevere in it). The second component is the belief in the ability to find solutions, resulting from perceiving oneself as a capable and resourceful person (pathway thoughts, pathways). Hope positively correlates with positive emotions and negatively with negative emotions; it is associated with a smaller number of negative thoughts, greater satisfaction with life and a lower level of depression and anxiety (Łaguna et al., 2005), including in clinical groups (Cui et al., 2020). It buffers the impact of stress on mental well-being (Bernardo et al., 2018). Hope is a positive correlate and predictor of school and academic achievement (Bryce et al., 2019).

According to Antonovsky (1987), we constantly encounter stressors—stimuli to which we do not have a ready, adequate adaptive response—but some of us cope with this experience quite well. In his opinion, effective coping with stress is determined by three factors: the types and levels of stressors, generalized immune resources and a sense of coherence. Generalized immune resources are properties of an individual—or a collective entity—that enable the avoidance of stressors or (when it is impossible) coping with the tension they generate (Pasikowski, 2000).

The ways of coping with stress play a special role in the course of a stress transaction. According to the transactional concept of stress, they are conditioned by the result of the secondary assessment—the ability to cope with stress (Lazarus and Folkman, 1984). They have also been included in the salutogenic model (Antonovsky, 1987).

In accordance with the assumptions of the salutogenetic model, however, the sense of coherence plays a key role in effective coping with stress. Sense of coherence is a global orientation of a person, expressing the degree to which a person has a dominant, persistent but dynamic sense of certainty that: (1) The stimuli flowing in the course of life from the internal and external environment are structured, predictable and explainable; (2) There are resources available to meet the demands of these stimuli; (3) For them, these requirements are a challenge worth the effort and commitment (Antonovsky, 1987). It consists of three components:

1. Comprehensibility—the ability to understand and cognitively evaluate reality as meaningful, information-ordered, coherent, clear and structured;
2. Manageability—the belief of individuals that they have means or resources, both personal and social, allowing them to actively influence the situation; a sense of competence in coping with a stressful situation, resulting from an adequate and realistic assessment of requirements and available resources;
3. Sense of meaningfulness—the conviction that it is worth engaging in challenging situations, related to the sense of meaning and value in one's own life. It plays a special role in shaping the sense of coherence.

The sense of coherence is often referred to as a meta-resource because:

- It influences the primary assessment of the stressor—people with a strong sense of coherence are less likely to assess the stimulus as stressful; they are convinced that they will cope with the situation (Pasikowski, 2000);
- It decides the strength of physiological and emotional reactions under the influence of a stressor (Kaczmarek, 2007);
- It conditions the undertaken remedial actions: people with a strong sense of coherence are more likely to focus on the problem and the available means to change the situation; they choose more appropriate coping strategies, assess the requirements and available resources more realistically, activate them more effectively, make better use of previous experience in coping with stress (Sȩk and Pasikowski, 2001);
- It also contributes to the development of other immune resources (Pasikowski, 2000).

Studies have shown a positive relationship between the sense of coherence and various measures of physical health (Eriksson and Lindström, 2005; Hakanen et al., 2007), including a positive mood (Sȩk and Pasikowski, 2001) and anxiety, depression, negative emotions, stress intolerance, aggression and self-aggression (Sȩk and Pasikowski, 2001; Eriksson and Lindström, 2005, 2007; Hakanen et al., 2007; Langeland et al., 2007). According to Mayer and Thiel (2014), the sense of coherence should be treated as the basic factor determining not only physical and mental health, but also high sports performance in elite athletes. This is confirmed by the results of the study of young Polish (Rutkowska and Wawer, 2012) and Hungarian (Sipos et al., 2015) athletes.

Both the immune resources and the sense of coherence affect how an individual copes with stress. The process of coping with stress consists of strategies—specific cognitive and behavioral efforts—aimed at mastering specific external and internal requirements, assessed by an individual as overburdening or exceeding their resources, undertaken in a specific stress transaction (Wrześniewski, 2000). They depend both on personality traits (e.g., optimism, self-esteem, and emotional reactivity) and individual preferences (including styles of coping with stress), other subjective features (gender, age, and education), the current psychophysical state of the individual, and the stressful situation itself (Strelau, 2000; Wrześniewski, 2000; Sȩk and Pasikowski, 2001; Heszen, 2014). The question arises as to how the athletes who prepare for the Olympic Games deal with the stress of the COVID-19 pandemic. Do their preferred strategies differ from those used by physical education students who engage in sports?

The figure shows the salutogenetic model in a synthetic way, highlighting in bold the variables that are of interest in our study.

The aim of the study was to compare the strategies of coping with stress during the COVID-19 epidemic of athletes involved in Olympic preparations and students of physical education, and to determine their depending on the variable gender. Predictor of strategies for coping with stress were also searched for among such explanatory variables as the components of the sense of coherence, hope for success, type of sports activity (elite players—PE students practicing sports) and gender.

The psychological situation of athletes were deprived of the possibility of training. The criterion for separating the studied group was played sport (regular physical activity) and above-average physical fitness. The experimental and control groups differ in terms of the level and nature of physical activity, although both groups are characterized by a higher level of these parameters than the general population. For both groups, the reduction in physical activity caused by the pandemic had more severe consequences than for the general population. For students, physical activity was required to complete practical classes at the university, while for athletes, being in shape and preparing for competitions. Moreover, changes to the dates of the Olympic Games increased the level of stress, as they are held once every 4 years and are extremely important sports competitions for each athlete.

**Figure 2:**
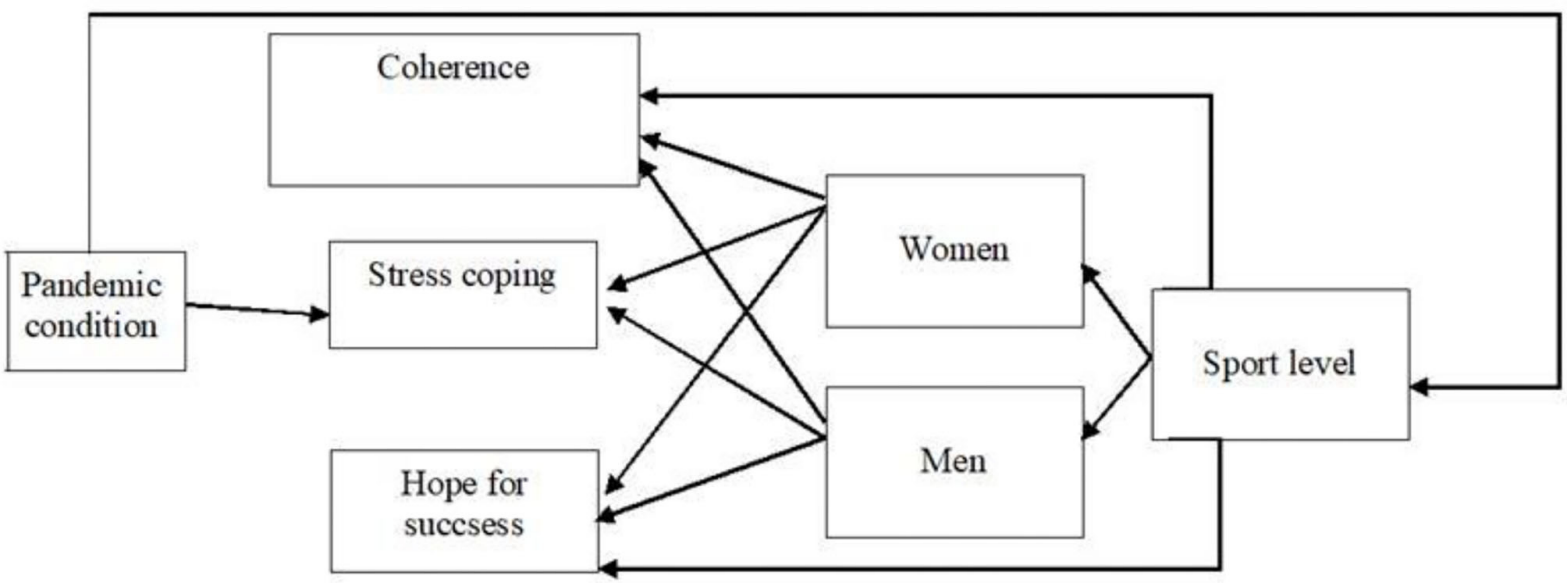
The model presenting the directions of dependencies in the research results.

There are many reasons to expect men and women to differ in their levels of stress and how they deal with it. Research results indicate that women are significantly more neurotic, perseverative and emotionally reactive, and consequently have a greater tendency to experience stress than men (Grossman and Wood, 1993). On the other hand, as shown by the results of studies on physically active people, physically active women and women practicing sports differ less from men in this respect (Messner, 1988). The unique situation of the pandemic provided an opportunity to see if the typical gender differences would be apparent in a group of physically active people experiencing stress from the pandemic and its consequences (Figure 2).

## Materials and Methods

### Test Subjects

The research involved two groups of people practicing sports at different levels. The first group consisted of 57 Polish potential Olympians aged between 18 and 45 (M = 26.61; SD = 5.562), including 29 women (52.7%) and 28 men (49.1%), practicing individual sports disciplines such as athletics, rowing, fencing, shooting, sport climbing, badminton, swimming, modern pentathlon, taekwondo, sailing, wrestling, canoeing, judo, cycling, equestrianism, and weightlifting. Their professional experience ranged from 4 to 25 years (M = 14.59; SD = 5.981). The second group consisted of 54 extramural students of physical education aged 19 to 40 (M = 25.69; SD = 5.908), including 26 women (48.1%) and 28 men (51.9%) who practiced recreational sports. The proportion of men and women in both groups did not differ significantly (χ^2^ = 0.083; *p* = 0.774).

Selection for the group was deliberate. The criterion for the inclusion of competitors in the study was to include athletes in Olympic preparation, which meant achieving an Olympic qualification or a good chance of obtaining it in the next year. The group of students was selected from among volunteers systematically undertaking recreational sports activities, in such a way that it was as close as possible to the group of athletes in terms of gender and age.

All subjects were of legal age. The survey was anonymous and voluntary. It was carried out in accordance with the principles of the Code of Ethics of the World Medical Association (Declaration of Helsinki). Manuscript is conformed to the Committee on Publication Ethics (COPE) and the International Committee of Medical Journal Editors (ICMJE) recommendations for ethics, as well as to the general Frontiers article requirements.

### Research Procedure

The research was conducted in the form of an on-line survey on the https://www.survio.com/pl platform in the period of April 7–28, 2020 during the COVID-19 pandemic. In Poland, in connection with the regulation of the Council of Ministers of March 31, 2020, the Ministry of Sport introduced a lockdown in participation in sports activities. Throughout the country, the use of outdoor sports facilities, indoor sports facilities (including stadiums, race tracks, swimming pools, gyms and other sports, and recreational facilities) was prohibited and the activities of sports teams and clubs were suspended. The lockdown also applied to the organization of sports events, the activities of athletes and sports referees. This lockdown lasted until May 4, 2020. All athletes participated in the study during the period of this lockdown. The average test time was ~25 min.

### Research Tools

Three standard psychological questionnaires were used (Table 1). The Stress Management Inventory (Mini COPE) (Carver, 1997) in the Polish adaptation of Juczyński and Ogińska-Bulik (2009) was used to study the coping strategy. It consists of 28 items and measures 14 strategies for coping with stress (two statements for each strategy): active coping, planning, positive re-evaluation, acceptance, sense of humor, turning to religion, seeking emotional support, seeking instrumental support, engaging in other activities, denial, discharging, taking psychoactive substances, ceasing activities, and blaming oneself.

**Table 1:** Variables used in the study.

**Variable**	**Variable**	**Indicator**
Stress coping	Dependent	The results of the questionnaire scales
Coherence	Independent	The results of the questionnaire scales
Hope for success	Independent	The results of the questionnaire scales
Physical activity (none)	Independent	Moderate level of activity/very high level of activity
Gender	modifying	Female/Male
Sport level	modifying	Medium/High
Pandemic conditions	Independent	Objective threatening situation

According to Parker and Endler (1994), these strategies can be grouped into three styles:

– task (active counseling, planning, and positive re-evaluation)
– emotional (acceptance, sense of humor, turn to religion, seeking emotional support, and seeking instrumental support)
– avoidance (dealing with something else, denial, unloading, taking psychoactive substances, stopping activities, and blaming oneself).

The respondents give answers on a scale from 0 = almost never to 3 = almost always. In the instructions, the respondents were asked to relate their answers to the current situation—the postponement of the Olympic Games. The Polish language version is characterized by a satisfactory split-half (0.86) and stability. The accuracy of the tool is confirmed by the results of the factor analysis.

The study of the sense of coherence was carried out using the Life Orientation Questionnaire (SOC 29, The Sense of Coherence Questionnaire) by Antonovsky (1987). In the Polish adaptation of Koniarek et al. (1993) it is a tool that allows one to estimate the general level of the sense of coherence, as well as the levels of its three components (i.e., the sense of comprehensibility, the sense of manageability and the sense of meaningfulness). It consists of 29 statements. While responding to them the respondent uses a 7-point Likert scale. The internal consistency of the entire scale is very high.

The Snyder et al. (1991) Hope for Success Questionnaire (KNS) in the Polish adaptation of Łaguna et al. (2005) was used to determine beliefs about the possibility of success. The tool contains two scales: the ability to find solutions and willpower. It also provides a summary indicator of hope for success. The factor structure is in line with the theoretical assumptions. The internal compliance index is estimated between 0.76 and 0.86.

### Statistical Analysis

In the first step, the basic descriptive statistics of the investigated quantitative variables were calculated along with the Kolmogorov-Smirnow (K-S) tests, checking the normality of distributions. In the case of the scales of the sense of comprehensibility and manageability, distributions close to the normal distribution were noted. For all other examined variables, the distributions different from the Gaussian distribution were noted. Since the variables did not meet the requirements of the normal distribution, the Mann-Whitney *U*-test was used to determine differences between the groups. Additional verification of the skewness of the distributions of these variables was done. Because it was in the range of +/− 2, it was decided that it was possible to perform a regression analysis.

The use of the stepwise regression method, this type of analysis was used because it has a mathematical basis. Purely mechanically, it enters the best predictors until none can be incorporated into the model anymore. It allows us to realistically obtain information which of the variables we analyze best allow us to predict the level of the dependent variable.

## Results

### Strategies for Coping With Stress, Sport Activity, and Gender

Regardless of gender, the surveyed athletes most often applied the strategy of positive re-evaluation, acceptance and planning (Table 2). The same was true for male students. For women studying physical education, the third rank was taken by engaging in other activities. The three strategies that are least used by athletes, regardless of gender and male students, are engaging in other activities, taking psychoactive substances, and denial. Female students used the self-blame strategy less than denial.

**Table 2:** Strategies for coping with stress depending on sports activity and gender.

	**AM amateur men**	**AW amateur women**	**SM sport men**	**SW sport women**	**Comparison**			
	**M ± SD**	**M ± SD**	**M ± SD**	**M ± SD**	**AM-SM**	**AW-SW**	**AM-AW**	**SM-SW**
Active coping	1.98 **±** 0.928	2.14 ± 0.934	1.39 ± 0.966	1.50 ± 0.872	**255.5**^**c**^	**226.5**^**b**^	360.5	341
Planning	**2.21 ± 0.726**	**2.29 ± 0.620**	**1.70 ± 1.003**	1.88 ± 0.909	276	284	393	322
Positive re-evaluation	**2.37 ± 0.647**	**2.65 ± 0.519**	**1.73 ± 0.799**	**2.17 ± 0.647**	**207.5**^**b**^	**218**^**b**^	296.5	257.5
Acceptance	**2.34 ± 0.667**	**2.65 ± 0.484**	**2.18 ± 0.627**	**2.31 ± 0.618**	326	**251**^**c**^	**288.5**^**c**^	324
Sense of humor	1.18 ± 0.710	1.14 ± 0.625	1.34 ± 0.594	1.19 ± 0.788	368	370.5	376.5	319.5
Turning to religion	0.68 ± 0.796	0.57 ± 0.764	0.75 ± 0.822	0.77 ± 1.051	373.5	359	373.5	344
Seeking emotional support	1.46 ± 0.882	2.07 ± 0.842	**1.70 ± 0.809**	1.88 ± 0.840	337.5	325.5	**242.5**^**b**^	309
Seeking instrumental support	1.11 ± 0.774	1.79 ± 0.861	1.32 ± 0.784	1.385 ± 0.804	346	278	**224**^**b**^	346
Engaging in other activities	1.07 ± 0.802	1.55 ± 0.880	1.52 ± 0.876	**1.92 ± 0.945**	284	287.5	**283.5**^**c**^	271.5
Denial	**0.16 ± 0.409**	**0.15 ± 0.465**	**0.43 ± 0.619**	0.60 ± 0.813	307	**252.5**^**b**^	378.5	330
Discharging	0.91 ± 0.681	1.20 ± 0.677	1.05 ± 0.497	1.31 ± 0.826	331	332	331.5	294.5
Taking psychoactive substances	**0.11 ± 0.416**	**0.33 ± 0.602**	**0.48 ± 0.645**	**0.35 ± 0.505**	**231.5**^**a**^	348	325.5	324
Ceasing activities	**0.10 ± 0.284**	**0.31 ± 0.618**	**0.30 ± 497**	**0.25 ± 0.381**	317	376.5	325	360
Blaming oneself	0.29 ± 0.584	0.41 ± 0.682	0.54 ± 576	**0.50 ± 0.616**	**260**^**b**^	336.5	354	338.5

a p < 0.001;

b p < 0.01;

c *p < 0.05. Bold values are statistically significant values p < 0.05*.

The nature of sports activity depending on the results more strongly than gender. Men involved in the Olympic preparations reported using the strategy of positive re-evaluation and active coping significantly more often than students, while they were less prone to self-blame and the use of psychoactive substances. Potential female Olympians used the strategy of active coping, positive re-evaluation and acceptance more often than students, while denial was used less often.

There are differences depending on the variable gender only in group of athletes. The competitors included in the Olympic preparations, when compared to their colleagues, showed a greater tendency to seek emotional and instrumental support, to engage in other activities and acceptance.

### Predictors of Stress-Coping Strategies

Step regression analyses were performed to determine the factors that would allow prediction of the frequency of using particular coping strategies. The components of the sense of coherence and hope for success, gender, age, and type of sports activity were introduced into the equation as explanatory variables. The dependent variables were the strategies of coping with stress. Table 3 shows the last steps in these analyses. No predictors for the frequency of using the sense of humor and discharge strategies were established.

**Table 3:** Sense of coherence and hope for success, gender, and type of sports activity as predictors of coping strategies.

**Strategy**	**Predictor**	**Beta**	***t, p***	***R^**2**^***	***F*; *p***
Active coping	Sense of meaningfulness	0.313	3.615; <0.001	0.199	14.546; <0.001
	Sports activity (A = 1; S = 0)	0.299	3.451; 0.001		
Planning	Strong will	0.516	5.510; <0.001	0.261	13.824; <0.001
	Sports activity (A = 1; S = 0)	0.209	2.445; 0.016		
	Sense of comprehensibility	−0.194	2.056; 0.042		
Positive re-evaluation	Sports activity (A = 1; S = 0)	0.390	4.558; <0.001	0.286	9.749; <0.001
	Gender (M = 1; F = 2)	0.197	2.401; 0.018		
	Strong will	0.231	2.338; 0.021		
	Sense of comprehensibility	−0.335	2.998; 0.003		
	Sense of manageability	0.265	2.287; 0.024		
Acceptance	The ability to find solutions	0.340	3.736; <0.001	0.158	7.804; <0.001
	Sense of comprehensibility	−0.243	2.631; 0.010		
	Sports activity (A = 1; S = 0)	0.212	2.316; 0.023		
Turning to religion	Sense of meaningfulness	0.193	2.049; 0.043	0.029	4.200; 0.043
Seeking emotional support	Strong will	0.312	3.485; 0.001	0.131	9.215; <0.001
	Gender (M = 1; F = 2)	0.202	2.262; 0.026		
Seeking instrumental support	Gender (M = 1; F = 2)	0.223	2.373; 0.019	0.041	5.632; 0.019
Engaging in other activities	Sense of comprehensibility	−0.259	2.859; 0.005	0.105	7.426; 0.001
	Gender (M = 1; F = 2)	0.238	2.627; 0.010		
Denial	Sports activity (A = 1; S = 0)	−0.268	2.953; 0.004	0.110	7.743; 0.001
	Age	−0.213	2.354; 0.020		
Taking psychoactive substances	Sense of comprehensibility	−0.360	4.004; <0.001	0.121	16.035; <0.001
Ceasing activities	Strong will	−0.349	3.875; <0.001	0.114	15.017; <0.001
Blaming oneself	Sense of manageability	−0.282	9.343; 0.003	0.071	9.343; 0.003

Among the analyzed strategies of coping with stress, the strongest model was obtained for a positive re-evaluation. Nearly 30% of the use of this strategy can be predicted based on five predictors. Competitors (rather than students), women, people who show strong conviction about having a strong will and a strong sense of manageability, but with a weak sense of comprehensibility, will be more likely to use this strategy.

The obtained model explains to a slightly lesser extent (26%) the frequency of using the planning strategy. All three predictors with the same sign were revealed previously: willpower and type of sports activity (positive predictors) and sense of comprehensibility (negative predictor).

Almost 20% of the willingness to use active coping strategies can be predicted based on the sense of meaningfulness and sports activity. It can be expected that this problem-focused strategy is more likely to be used by athletes and those with a strong sense of meaningfulness.

The variable explained in over 15% is the propensity to use the acceptance strategy. It can be expected that it will be used more often by athletes than students and people with high confidence in the ability to find solutions, but with a low sense of comprehensibility.

In the remaining models, the coefficients of determination had lower values (0.13−0.03). Women seem to be more inclined to seek emotional and instrumental support. The predictor of the use of avoidance strategies (engaging in other activities, taking psychoactive substances) is a poor sense of comprehensibility. People with a weak belief in the ability to initiate an action and its implementation seem to be prone to stopping an action (belief in having a strong will). Students and younger respondents are more likely to use denial.

## Discussion

Dealing with stress is of interest to psychologists, as it is the effectiveness of this process that largely determines the health costs of a stress transaction (Antonovsky, 1979). What specific actions people take when faced with stress depends both on the characteristics of the subject and the situation (characteristics of the stressor). A pandemic situation is a strong external stressor affecting the whole society, which can be treated as an extreme event [i.e., one that causes stress for all people experiencing it, regardless of the subjective features and perception of the stressful situation (Heszen, 2014)]. It is beyond the control of the individual and it changes everyday life in a radical way. Otherwise was in the newest study about athletes and pandemic which results showed that chess players significantly decreased physical activity per day while increased chess practice during the confinement period. Amateur players showed a significantly higher level of social alarm than professional and high-performance players. Moreover, professional players showed higher values of extraversion than high-performance players and amateur players. In neuroticism, professional players showed higher values than high-performance players. In addition, the professional players showed higher scores in psychological inflexibility than competitive players (Fuentes-García et al., 2020).

The elite athletes in preparation for the Olympic Games and physical education students practicing sports coping with the stress of the pandemic, regardless of gender, most often used the acceptance strategy. The acceptance strategy is about accepting the situation (recognizing the reality of the stressor) and learning how to live in it without actively trying to change the situation. The mini-COPE questionnaire has no standards for the Polish population. Therefore, we can only refer the results of our study to the values obtained by the authors of the tool adaptation (Juczyński and Ogińska-Bulik, 2009). The acceptance strategy came third in terms of the frequency of use by adult Poles and only seventh for students. Thus, both athletes and PE students, in a pandemic situation, used the acceptance strategy more often than the groups studied by Juczyński and Ogińska-Bulik (2009). Acceptance of the pandemic situation seems to be an appropriate remedial strategy. First, the individual assesses the situation as stressful (exceeding resources) (i.e., he or she is confronted with the fact that it poses a real threat to health and life). Second, he or she is aware of the limited possibilities for active coping. If it is not possible to change the situation, adaptation requires an autoplastic adaptation (i.e., a change in the subject).

The second rank in terms of the frequency of use, regardless of gender and sports activity, was taken by the strategy of positive re-evaluation, which in nationwide surveys came fourth (adults) and fifth (students) (Juczyński and Ogińska-Bulik, 2009). In dealing with the stress of the pandemic, the elite athletes and students of physical education we surveyed more often tried to perceive the situation in a more positive light. In the studies on the mini-COPE factor structure, the strategy of positive re-evaluation was included in the active coping factor. However, it differs from active behavioral coping as it takes place in the cognitive realm. It provides an example of meaning-focused coping (Folkman and Moskowitz, 2006). The results of previous studies confirm that the strategy of positive re-evaluation is associated with a lower intensity of stress, especially when the individual is unable to influence the change of the stressful situation. In the event of the COVID-19 pandemic, the stressful situation is partly out of control. Research on coping strategies during the COVID-19 epidemic shows that coping with stress using positive strategies is a predictor of lower stress levels and mental well-being (Fredrickson and Joiner, 2002).

In the group of athletes involved in the preparations for the Olympic Games, the planning strategy was ranked third, as was the positive reevaluation that made up the active coping factor. Planning is thinking about how to deal with a stressor. It is cognitive in nature and occurs during secondary evaluation. As a result, the individual takes specific, behavioral remedial actions. In nationwide studies, planning was the strategy most often undertaken by adults, slightly less often than active coping and second only to active coping in the case of students (Juczyński and Ogińska-Bulik, 2009). Active coping came fourth among the elite athletes. The research was conducted by us in the second month of the COVID-19 epidemic in Poland, when athletes planned what they would do rather than take specific remedial actions. This hierarchy of coping strategies seems to serve well for adaptation to the stress of a pandemic.

Athletes, regardless of gender, and male students were the least likely to use the cessation strategy (abandonment of efforts to achieve the goal), taking psychoactive substances and denial (active cognitive rejection of the stressful situation). Female students used the self-blame strategy less than denial. All strategies are avoidant in character and are usually considered ineffective. It is also worth noting that the frequency of using all these strategies was lower than in the national studies (Juczyński and Ogińska-Bulik, 2009). Our results show that during the pandemic, elite athletes and students most often used strategies that can be considered effective and aimed at good adaptation to an extreme stressor causing a threat to health and life.

The elite athletes differed from the university students in the frequency of coping strategies. Regardless of their gender, they used more active coping and re-evaluation strategies. Also, in the studies by Nicholls et al. (2007) highly qualified athletes reported more frequent use of problem-focused strategies (planning, blocking and visualization) and greater effectiveness in coping than lower-level athletes.

Confrontation with a stressor, both in the behavioral and cognitive form, is a prerequisite for managing stress in controllable situations (Heszen, 2014). Athletes most often face the stress of sports competition. In this case, they prefer task-oriented strategies (Hofseth, 2016; Litwic-Kaminska and Izdebski, 2016). Athletes researched by Crocker and Graham (1995) also most frequently used problem-focused strategies: effort, planning, and active coping. Research results confirm that problem-focused strategies better serve the level of athletic performance than strategies focused on emotions or avoidance (Folkman, 1992; Nicholls et al., 2012). Problem-focused coping, including active coping, was associated with positive affect and higher self-esteem of athletic performance (Ntoumanis and Biddle, 2000). Athletes deal with non-sport related stressors—such as ending a sports career—in a similar way. They most often use the strategy of acceptance, positive re-evaluation, planning and active coping (Grove et al., 2008).

Athletes accustomed to actively coping with stress (Polman, 2012) probably did not change their preferences, using behavioral and cognitive confrontation strategies effective against stress, including in the face of stress related to the COVID-19 pandemic.

Athletes of both sexes reported the use of the denial strategy less frequently than students. Men practicing sports additionally used psychoactive substances less often and less often blamed themselves. Thus, athletes used problem-focused strategies (both cognitive and behavioral) more often, and avoidance strategies less frequently. Men practicing sports clearly used strategies indicating helplessness in confronting the stress of a pandemic less frequently. These differences are confirmed by the results of the regression analysis. The type of sports activity turned out to be a predictor of the frequency of using the five remedial strategies. Athletes should be expected to be more likely to use active coping strategies (active coping, planning, and positive re-evaluation) and acceptance, while physical education students seem to be more prone to denial.

It is believed (Heszen, 2014) that cognitive and behavioral avoidance strategies are adaptive in uncontrolled situations. In a situation where an athlete has limited ability to change the situation, avoidance strategies may be more effective (Carver et al., 1989). However, they are not widely used by athletes (Nicholls et al., 2009). Is the individual able to control a pandemic situation? They cannot control its course, but they can control their own behavior and reduce the risk of infection. It can therefore be concluded that the coping patterns revealed by the athletes served well for adaptation to the COVID-19 pandemic situation.

There are differences depending on the variable gender in the group of athletes which are more strongly than in the case of students. Competitors included in the Olympic preparations, as compared to their colleagues, reported more frequent searching for emotional and instrumental support, but also using the strategy of engaging in other activities and acceptance. The results of the regression analysis were similar: female gender was found to be a positive predictor of using the strategy of seeking emotional and instrumental support, engaging in other activities and positive re-evaluation. Crocker and Graham (1995) also found that female athletes were more likely to seek emotional support than male athletes. Somewhat different results were obtained by Nicholls et al. (2007)—women practicing sports more often than men applied problem-focused strategies (e.g., planning, communication, and technique-focused coping). The authors themselves note, however, that their results clearly differ from the results of previous studies.

It can be assumed that, at least in part, the differences we identified were due to the greater willingness of women to disclose remedial actions. Moreover, the search for support and help is part of the social role of women. The lower tendency of men to seek or use the available social support in order to cope with stress is explained, among other factors, by fear of being negatively judged (Sirois and Kitner, 2015). Women may also be more likely to engage in surrogate activities and to postpone decision-making and realization over time, due to different patterns of emotional self-regulation (Doyle and Paludi, 1998). Summarizing the results of research on the coping process Lazarus (1993a,b) stated that, contrary to popular beliefs about gender differences in a particular stressful situation, women and men exhibit a very similar coping pattern. Also Crocker and Graham (1995) did not confirm the expected gender differences (more frequent use of problem-focused coping by men) in their study of athletes. Research results are not consistent, so more research is needed on the differences in how women and men deal with various stressors. The aim of Clemente-Suárez et al. (2020) was to analyze the effect of psychological profile, academic schedule, and gender in the perception of personal and professional threat of Olympic and Paralympic athletes facing the 2021 Tokyo Olympiad in the actual COVID-19 crisis. Neuroticism and psychological inflexibility presented the greatest negative feelings for female athletes and the perception that quarantine would negatively affect their sports performance.

The female athletes we surveyed were more similar to the male respondents than female students in terms of the hierarchy of coping strategies. Women who are involved in competitive sports often have features attributed to men. Androgynous people (combining typical male and female characteristics) reveal greater psychological resources, allowing them to deal with stress more effectively (Lipińska-Grobelny and Gorczycka, 2011).

The models turned out to be the strongest in relation to the strategies most often used by athletes and students, which in the research on the adaptation of the tool to Polish conditions were included in the factor of active coping (positive re-evaluation, planning and active coping). The models were somewhat weaker for the strategies constituting the helplessness factor, which were used the least (substance use, stopping action, and self-blame). No predictors for the frequency of using the sense of humor and discharge strategies were established.

When analyzing individual explanatory variables, we can observe that the sense of comprehensibility was a negative predictor of using cognitive strategies (planning, positive re-evaluation, and acceptance) and avoidance strategies (engaging in other activities, using psychoactive substances). The low ability to understand and cognitively evaluate reality as meaningful, information-ordered, coherent, clear and structured, therefore, intensifies the use of these strategies. The research involving Polish students (Pasikowski, 2000) also established negative, weak relationships between the sense of comprehensibility and cognitive strategies: logical analysis, cognitive avoidance and acceptance. Perhaps a positive re-evaluation—giving a positive meaning to an event—involves restructuring the problem so that it is easier to assimilate cognitively. Also, creating an action plan allows one to structure the problem situation. People who find the reality unstructured and coherent are more prone to cognitive analysis. Acceptance, in turn, involves recognizing reality as it is, even when it is difficult to “embrace it with the mind.” As a result of cognitive reevaluation, cognitive adaptation to threatening events occurs (Schwarzer and Knoll, 2003). It can be assumed that when cognitive strategies are not effective, people with a low sense of comprehensibility use avoidance strategies such as using psychoactive substances or taking care of something else. In Pasikowski's (2000) research, the sense of comprehensibility also negatively correlated with emotional discharge. In our study, however, it did not allow us to predict the frequency of using an emotional strategy.

It can be assumed that when cognitive strategies are ineffective, people with a low sense of comprehensibility use avoidance strategies, such as using psychoactive substances or engaging in other activities. In Pasikowski's research (2000), the sense of comprehensibility was also negatively correlated with emotional discharge. Our study, however, did not allow us to predict the frequency of using an emotional strategy.

The other components of the sense of coherence seem to predict to a lesser extent that the coping strategies of the COVID-19 pandemic will be used by elite athletes and physical education students. The sense of meaningfulness was a positive predictor of active coping (taking active steps toward removing or avoiding the source of stress or mitigating its consequences). To act, one needs a sense of meaningfulness—the conviction that it is worth engaging in challenging situations. This dimension of the sense of coherence was positively correlated with taking actions to solve the problem by Polish students surveyed by Pasikowski (2000).

Interestingly, people with a strong sense of meaningfulness can be expected to turn to religion more often, which can provide emotional support. Perhaps in the case of some of the respondents, faith is strongly related to the sense of meaningfulness and value of their own life. Carver et al. (1989) do not rule out that for deep believers, turning to religion may be a way of actively dealing with the problem. Some researchers use religious (or, more broadly, spiritual) coping (Folkman and Moskowitz, 2004) treated as a separate form of coping with stress. It is especially useful in the face of an uncontrollable source of stress and helps to find meaning in unfavorable events and suffering. This strategy is relatively rarely used by the athletes and students we surveyed; it ranks 10th in the hierarchy, similar to the adult population studied by Juczyński and Ogińska-Bulik (2009).

Dimensions of hope for success were more predictive of coping strategies than sense of manageability (a negative predictor of self-blame and a positive predictor of positive re-evaluation). The belief in having willpower was a positive predictor of active cognitive strategies for planning and positive re-evaluation and seeking emotional support and a negative predictor of disengagement. Contrary to the results of previous studies, the hope for success did not allow prediction of active coping with the stress of the COVID-19 pandemic. In the research of Polish flood victims (Chmielewska and Trzebiński, 2004), the hope for success was associated with taking specific actions (personal involvement in the reconstruction of a house, applying for a bank loan, seeking help from people and institutions). People taking active steps to deal with unemployment were characterized by a stronger hope for success, which allowed the entrepreneurial intention to be anticipated (Łaguna, 2005).

There is, however, a significant difference between experiencing the negative effects of a flood and unemployment (harm/loss situation) and a pandemic (emergency, largely uncontrollable situation).

It is worth noting that the explanatory variables included in the study made it possible to predict the frequency of using remedial strategies to a limited extent; the coefficients of determination ranged from 0.286 to 0.029, and in two cases no predictors were established. The sense of coherence and hope for success, of course, do not exhaust the subject's properties that affect how an individual deals with stress in a specific situation. The research results confirm the role of personality traits in a broad sense, including temperament, beliefs about the world and oneself, and styles of coping with stress (Heszen, 2014).

## Limitations

Our work is obviously not free from limitations. Its weak point is the small number of respondents. It should be remembered, however, that the criterion for inclusion in the research was the respondent athlete's participation in the preparations for the XXXII Olympic Summer Games. We used a universal tool for studying remedial strategies—mini-COPE, adding to the instructions a reference to the pandemic situation. Perhaps it would be more appropriate to use a scale designed to study strategies for coping with catastrophic stressors. However, we did not have such a tool. The lack of standards for mini-COPE in the Polish population made it impossible to precisely interpret the results; they could only be related to the average results of adults and students obtained in the process of adapting the scale to Polish conditions. Based on the salutogenetic model, we focused on the importance of the sense of coherence as a potential factor determining how people deal with stress. Among the many potential immune resources, we chose the hope for success, which we found to be an important construct for athletes striving for success—the Olympic qualification. Of course, this does not exhaust all possible resources. In the online survey, the risk of discouraging participants with too many questions is greater than in the case of auditorium surveys. This forced the limitation of the research tools used and forced us to make a choice.

## Conclusions

1. Elite athletes and physical education students involved in sports most often coped with the stress of the COVID-19 pandemic using active coping strategies at the cognitive and behavioral level.
2. The elite athletes confirmed greater readiness to use them than students of physical education.
3. The strategies for coping with stress caused by the COVID-19 pandemic differ depending on the sport level variable, even more than depending on the gender variable.
4. The relationship of the sense of coherence (mainly the sense of comprehensibility) and hope for success treated as a generalized immune resource with coping strategies in the case of the COVID-19 pandemic postulated by Antonovsky was confirmed.

## Data Availability Statement

The raw data supporting the conclusions of this article will be made available by the authors, without undue reservation.

## Ethics Statement

The studies involving human participants were reviewed and approved by Senacka Komisja Etyki Badań Naukowych AWF WARSZAWA. The patients/participants provided their written informed consent to participate in this study.

## Author's Note

Regulation - On the establishment of certain restrictions, orders and bans in connection with an epidemic (Journal of Laws, item 566), on the basis of the Act of March 6, 2018 - Law of entrepreneurs and other entities, activities related to sports, entertainment and recreation (included in the Polish Classification of Activities in section 93.0 and in subclass 96.04.Z), access date 15.06.20.

## Author Contributions

All authors listed have made a substantial, direct and intellectual contribution to the work, and approved it for publication.

## Conflict of Interest

The authors declare that the research was conducted in the absence of any commercial or financial relationships that could be construed as a potential conflict of interest.
